# Solubility Difference between Pectic Fractions from Creeping Fig Seeds

**DOI:** 10.3390/polym11010159

**Published:** 2019-01-17

**Authors:** Ri-si Wang, Xiao-hong He, Hong Lin, Rui-hong Liang, Lu Liang, Jun Chen, Cheng-mei Liu

**Affiliations:** 1 State Key Laboratory of Food Science and Technology, Nanchang University, 235 Nanjing East Road, Nanchang 330047, China; ncuskwangrisi@163.com (R.-s.W.); hexiaohongmaimai@163.com (X.-h.H.); liangruihong@ncu.edu.cn (R.-h.L.); 18679113010@163.com (L.L.); liuchengmei@ncu.edu.cn (C.-m.L.); 2 Jiangxi Academy of Forestry, Nanchang 330032, China; ncuskxiashiqi@163.com

**Keywords:** creeping fig seeds, low methoxyl pectin, pectic fractions, solubility

## Abstract

Crude water-extracted pectin (WEP) isolated from creeping fig seeds were mainly fractionated into WEP-0.3 and WEP-0.4 fractions. Fractions were confirmed to be nonstarch, nonreducing sugars, nonpolyphenols and protein-unbounded acidic polysaccharides. Interestingly, a significant difference in solubility was found between WEP-0.3 (higher solubility than WEP) and WEP-0.4 (remarkably insoluble), which was consistent with the amorphous and porous sponge-like structure of WEP-0.3 as well as the crystalline and dense rod-like state of WEP-0.4. However, the result of the FT-IR spectra was contradicted by the solubility of WEP-0.4, which possessed the lowest degree of methoxylation and ought to possess the highest solubility. Through mineral analysis, a considerably high content of Ca^2+^ was found in WEP-0.4, suggesting that the low solubility of WEP-0.4 was probably attributable to the formation of microgels during dialysis. Therefore, metal divalent cations in the dialysate were suggested to be depleted for the dialysis of low methoxyl pectin.

## 1. Introduction

Pectin is a complex heteropolysaccharide that ubiquitously exists in the cell wall of land plants. It has been extensively applied to the food, cosmetic and pharmaceutical industries owing to its unique functional properties of gelling, emulsifying and stabilizing [1,2]. The structure of a pectin macromolecule encompasses a linear backbone of α-1,4-linked d-galacturonic acid (GalA) residues in which the carboxyl groups of the GalA can be free or methyl-esterified (homogalacturonan blocks, HG) and some regions attached to HG blocks which have side chains (rhamnogalacturonan blocks, RG) [3,4]. Commonly, pectin is categorized according to the degree of methoxylation (DM) as high methoxyl pectin (HMP, DM > 50%) or low methoxyl pectin (LMP, DM < 50%). The gels obtained from LMP are softer, more spreadable and relatively independent of pH as well as low-calorie without the addition of sugar compared to HMP gels [5,6]. It is also stated that LMP has become increasingly prevalent as a fat replacer in dietetic foods and has spurred special attention in the food industry [7]. However, pectin produced at the industrial level is mostly HMP, which is typically obtained by using citrus peel, apple pomace and sugar beet pulp as raw materials [8]. Meanwhile, LMP is usually manufactured from HMP via acid de-methoxylation [9], alkali de-methoxylation [10], pectin methyl esterase de-methoxylation [11] and ammonia in alcohol or via concentrated aqueous ammonia de-methylation and amidation [12]. A better alternative to produce LMP for food and pharmaceutical application is to explore local endogenous plant species whose cell walls are enriched with LMP.

Creeping fig (*Ficus pumila* Linn) of the Moraceae family is a perennial and scandent shrub plant that grows vigorously on mountain sides and broken walls, as well as along the trunks of tall trees in China, Japan and some other countries. The seeds of this plant, which are rubbed and squeezed in water to yield a mucilaginous water extract, have a long history of being used to make a summer drink named “Liang fen” for local people. In our previous study, the main component of the water extract was found to be LMP. This pectin has the peculiarity of being extracted more easily when compared with other industrial pectin resources, such as citrus peel and apple pomace, because it is located in a transparent layer on the surface of seeds [13].

Polysaccharides are often fractionated to better illustrate their structure and physicochemical properties, and to facilitate the ready utilization of such polysaccharides in food and other industries. In this study, water-extracted pectin (WEP) isolated from creeping fig seeds was fractionated into WEP-0.3 and WEP-0.4 by anion-exchange chromatography. It was surprising to find that these two fractions exhibited a significant difference in solubility. Therefore, a series of experiments, including environmental scanning electron microscopy (ESEM), X-ray diffraction, FT-IR spectroscopy and mineral analysis were carried out to explore the reasons for the solubility difference.

## 2. Materials and Methods

### 2.1. Materials

Galacturonic acid was procured from Sigma Aldrich Co., Shanghai, China. Bovine serum albumin was supplied by Bioleaf Biotech Co. Ltd. (Shanghai, China). Coomassie brilliant blue G-250 was procured from Solarbio Biotech Co. Ltd. (Shanghai, China). All chemical reagents used in the experiments were analytical grade.

### 2.2. Extraction of WEP

The preparation of plant materials and extraction of WEP were carried out according to our previous method [13]. Briefly, dried creeping fig seeds were extracted thrice with distilled water at 25 °C for 30 min under magnetic stirring where a solid/liquid ratio of 1:20 (*w*/*v*) was applied. The extracts were then filtered to remove the solid sediments and precipitated by the addition of ethanol to obtain a final concentration of 50% (*v*/*v*). Thereafter, the precipitates were washed successively with ethanol/water mixtures of the following volume ratios: 70/30, 80/20, 90/10, as well as absolute ethanol. The obtained pectins were lyophilized and stored in hermetically sealed glass bottles.

### 2.3. Fractionation of WEP by DEAE Fast Flow Chromatography

The fractionation of WEP was performed by using anion-exchange chromatography according to the method of Sun et al. [14] with some modifications. A total of 10 mg crude extract was re-dissolved in 20 mL distilled water and centrifuged at 4800 rpm for 20 min. The supernatant was further purified by using an anion-exchange column (25 cm × 2.6 cm) of DEAE FAST FLOW (Sigma Aldrich, Shanghai, China) equilibrated with distilled water. The column was sequentially eluted with four column volumes distilled water, four column volumes 0.1 M NaCl, four column volumes 0.2 M NaCl, eight column volumes 0.3 M NaCl, eight column volumes 0.4 M NaCl and four column volumes 0.5 M NaCl. A total of 6 mL/tube of eluent was collected at a flow rate of 0.4 mL/min. The polysaccharide concentration of the eluent in each tube was quantified by the phenol-sulphuric acid method [15]. At the same time, the concentration of galacturonic acids was determined according to the method described by Blumenkrantz et al. [16]. The eluents containing reducing sugar and those using the same eluent in the process of elution were combined individually. Collected fractions were placed in a regenerated cellulose bag filter (MWCO 8000, Spectrum Laboratories Inc., Rancho Dominguez, CA, USA) and successively dialyzed against tap water and distilled water for 1 day. Subsequently, fractions were further concentrated by a vacuum rotary evaporator at 50 °C. Finally, some fractions were used for the qualitative analysis of some general physicochemical properties, and the others were lyophilized and stored in a desiccator at room temperature.

### 2.4. General Physicochemical Properties Analysis and Solubility

General physicochemical properties were assayed by using the following methods: color observation, iodination reaction, Fehling’s test, FeCl_3_ reaction, carbazole reaction, full wavelength scanning and Coomassie brilliant blue reaction [17].

The water solubility of WEP and its fractions was determined by the method of Monsoor et al. [18]. Samples (1.0 g) were suspended in 100 mL of distilled water and incubated at room temperature with constant stirring (500 rpm). Afterward, the suspensions were centrifuged at a specific time (4800 g, 15 min), and insoluble pectin portions were collected, freeze-dried and then dried at 50 °C in the oven until constant weight was achieved. The percentage of solubility was calculated as follows:

$$\%\;\mathrm{Solubility}=\frac{W_{S}-W_{i}}{W_{i}}$$

where W_i_ is defined as initial weight of the samples, and WS is the weight of the dried insoluble pectin portions.

### 2.5. X-ray Diffraction (XRD) Analysis

The X-ray diffraction patterns of samples were recorded with an X-ray diffractometer (D8-focus, Bruker, Karlsruhe, Germany) operated at 40 kV and 40 mA with Cu Kα radiation. The samples were scanned over the range of 5° to 60° diffraction angle (2θ) with a step size of 0.02° (2θ) and a counting time of 0.2 s/step.

### 2.6. Environmental Scanning Electron Microscopy (ESEM) Analysis

Samples were mounted onto a circular specimen stub using double-sided tape and observed using an environmental scanning electron microscope (ESEM) (Quanta 200F, FEI Deutschland GmbH, Kassel, Germany) at 30 kV voltage with a 3.0 spot size. Low vacuum mode was used while operating the ESEM.

### 2.7. FT-IR Spectroscopy

The FT-IR spectra of pectin were recorded on a Nicolet 5700 spectrometer (Thermo Co., Madison, WI, USA). The dried samples were ground with spectroscopic-grade KBr powder (Sigma Aldrich, Shanghai, China) and pressed into pellets for spectra measurement in the frequency range of 4000–400 cm^−1^ [19]. The data were collected and plotted as transmittance (%) as a function of the wave number (cm^−1^) and analyzed with Ominic 7.2 software (Spectra-Tech Inc., Madison, WI, USA).

The degree of methoxylation (DM) was calculated by using FT-IR spectra based on the method of Gnanasambandam et al. [20]. A linear correlation was established between the ratio of the peak area at 1740 cm^−1^ (υ_(C=O)COOMe_) over the sum of the peak areas at 1740 cm^−1^ and 1630 cm^−1^ (υ_as (COO)_) based on pectin standards with known DMs. After that, the peak area of the ester carbonyl of sample was fit to the curve to calculate its DM.

### 2.8. Mineral Analysis

Mineral composition and content were determined by using inductively coupled plasma–atomic emission spectrometry (ICP–AES) (Optima 5300DV, Perkin–Elmer Corporation, Waltham, MA, USA). About 1 gram of pectin was accurately weighed into a 100-mL beaker, and 20 mL mixed acid containing nitric acid/perchloric acid (3:1) was added. The sample was heated on a heating plate for 40 min, and then quantitatively transferred to a volumetric flask with a fixed capacity of 100 mL. The supernatant was used for the mineral analysis.

### 2.9. Statistical Analysis

All the experiments were conducted in triplicate. Statistical analysis was carried out using SPSS (version 16.0, Chicago, IL, United States). The results are expressed as means ± standard deviations and were compared using the Tukey test at a 5% confidence level.

## 3. Results

### 3.1. Fractionation of WEP 

**Figure 1:**
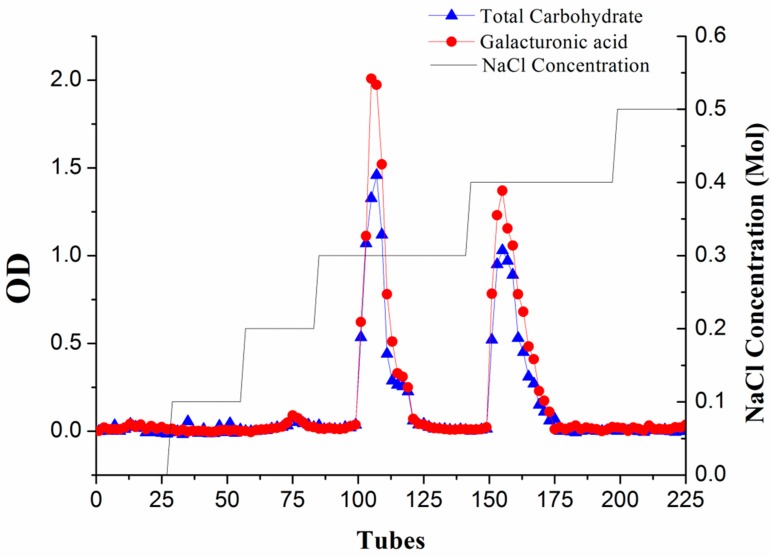
The profile of water-extracted pectin (WEP) on anion-exchange chromatography was eluted with distilled water and a stepwise gradient of NaCl aqueous solutions (0.1–0.5 M NaCl), detected by the phenol-sulphuric acid method at 490 nm and the m-hydroxybiphenyl method at 525 nm.

The charge heterogeneity of WEP can be observed from the elution curve of the anion-exchange column (Figure 1). Two major fractions, respectively separated at 0.3 M and 0.4 M NaCl (WEP-0.3 and WEP-0.4), were obtained, accounting for 49.73% and 42.28% of WEP, respectively. A small number of fractions were eluted at a NaCl concentration of 0.2 M, while trace sugars were found when using distilled water, 0.1 M and 0.5 M NaCl as eluent, implying that WEP was mainly composed of acidic polysaccharides. WEP-0.3 represented 61.11% and WEP-0.4 accounted for 38.89% of recovered sugars. In addition, WEP-0.3 and WEP-0.4 showed one main uronic acid peak, indicating that both fractions were pectic polysaccharides. WEP-0.3 was eluted at a lower ionic strength, reflecting its higher degree of esterification in comparison to WEP-0.4.

### 3.2. Solubility

**Figure 2:**
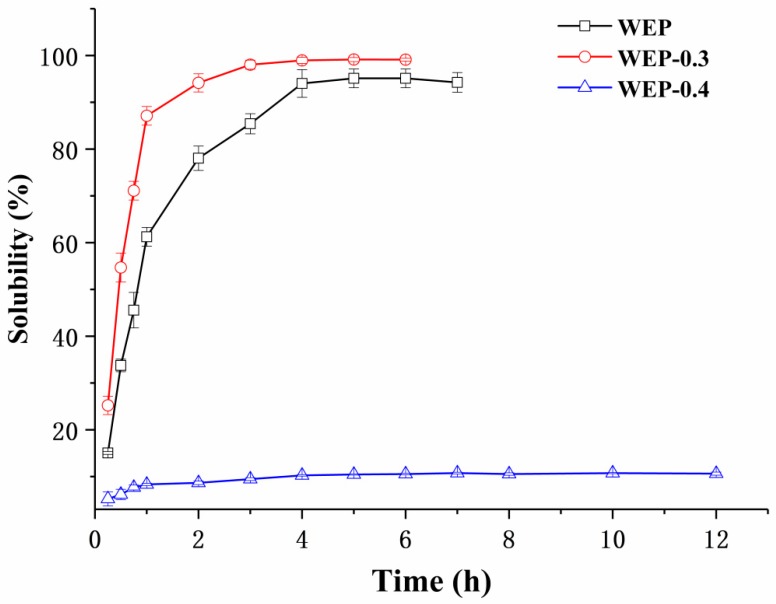
The solubility of WEP, WEP-0.3 and WEP-0.4 in water as a function of dissolution time.

Pectin is usually provided in a powdery form and must be dissolved in an aqueous solution prior to use. Its solubility and dissolution kinetics are thus pivotal specifications in a large number of technological application processes. Figure 2 shows the solubility of WEP, WEP-0.3 and WEP-0.4 as a function of dissolution time. For WEP, it was steadily soluble in water and reached its maximum degree of solubility (around 95%) after 4 hours. Interestingly, its two fractions exhibited a significant difference in solubility. WEP-0.3 was almost completely dissolved in water after 3 h. Moreover, it showed a better solubility than WEP at any time point within 12 h. In contrast, WEP-0.4 was dissolved slowly and a majority of the counterpart (about 89.5%) was insoluble in water. This phenomenon was extremely intriguing, and previously there was no concern about solubility differences between polysaccharide fractions after fractionation. In order to explain the varied solubility of WEP fractions, a series of experiments, including general physicochemical properties analysis, X-ray diffraction analysis, environmental scanning electron microscopy, FT-IR characterization and mineral analysis were carried out.

### 3.3. General Physicochemical Properties Analysis 

A general physicochemical properties analysis was performed to check the possibility of the presence of insoluble materials in WEP and its fractions and the results are listed in Table 1. A Carbazole reaction was used to assay galacturonic acid and it corroborated that both WEP-0.3 and WEP-0.4 were pectic polysaccharides. The color of WEP appeared to be light yellow, while the two fractions were both snowy white. It was conceivable that some water-soluble pigments were removed during the process of fractionation. Negative reactions to the iodination, Fehling’s and FeCl_3_ reaction were presented, thus revealing the absence of starch, reducing sugars and polyphenol in WEP and its fractions. Coomassie Brilliant blue assays suggested that protein was present in WEP. Nevertheless, no protein was found in WEP-0.3 and WEP-0.4 fractions. This result implied that the protein in WEP was unbounded, which could be detached after fractionation. The full wavelength scan also confirmed that protein was not present in WEP-0.3 and WEP-0.4 fractions. Therefore, the solubility difference between WEP-0.3 and WEP-0.4 was not attributable to the presence of insoluble materials, but due to other reasons.

### 3.4. X-ray Diffraction Analysis

**Figure 3:**
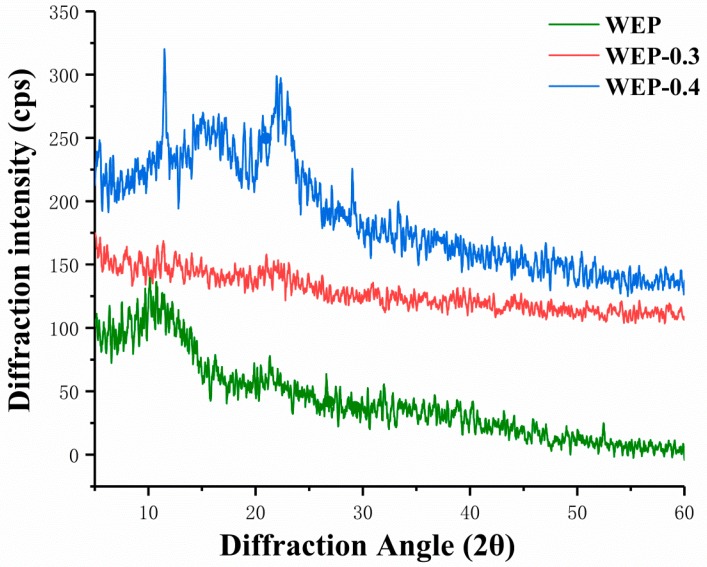
X-ray diffraction patterns of WEP and its fractions. (**a**) WEP; (**b**) WEP-0.3; (**c**) WEP-0.4.

It was reported that amorphous solids generally have higher solubility and higher dissolution rate because they have higher free energy than corresponding crystals [21]. Therefore, X-ray diffraction was used to provide amorphous or crystalline information of samples, and results were displayed in Figure 3. WEP-0.3 was amorphous structure showing no distinct crystal peaks (Figure 3). In amorphous regions of polysaccharides, molecules or chain segments are in a disordered or disorganized distribution, and only partial intermolecular forces and hydrogen bonding were satisfied [22]. Hence, substantial unsatisfied hydrogen bonding positions were present in amorphous WEP-0.3 which can hydrate giving rise to high solubility. Whereas, some crystal peaks appeared in WEP and WEP-0.4 (Figure 3). The crystalline nature may be because pectins constitute a highly complex and heterogeneous group of polysaccharides, which content distinctive covalently linked domains that differ in their molecular organization and crystallinity [23]. Sharma et al. [24] and Jiang et al. [25] also observed crystalline nature of pectin. According to Kravtchenko et al. [26], dissolution is a conjugate process which involves in two phenomena, including the solvent penetration and the polymer dissolution. It is most probable that the existing of crystal inhibited the penetration of solvent resulting in having difficulty with dissolution. The higher the degree of crystallinity, the inferior solubility of samples. WEP-0.4 exhibited more distinct crystalline structure, thus exhibiting the most inferior solubility.

### 3.5. Environmental Scanning Electron Microscope (ESEM)

**Figure 4:**
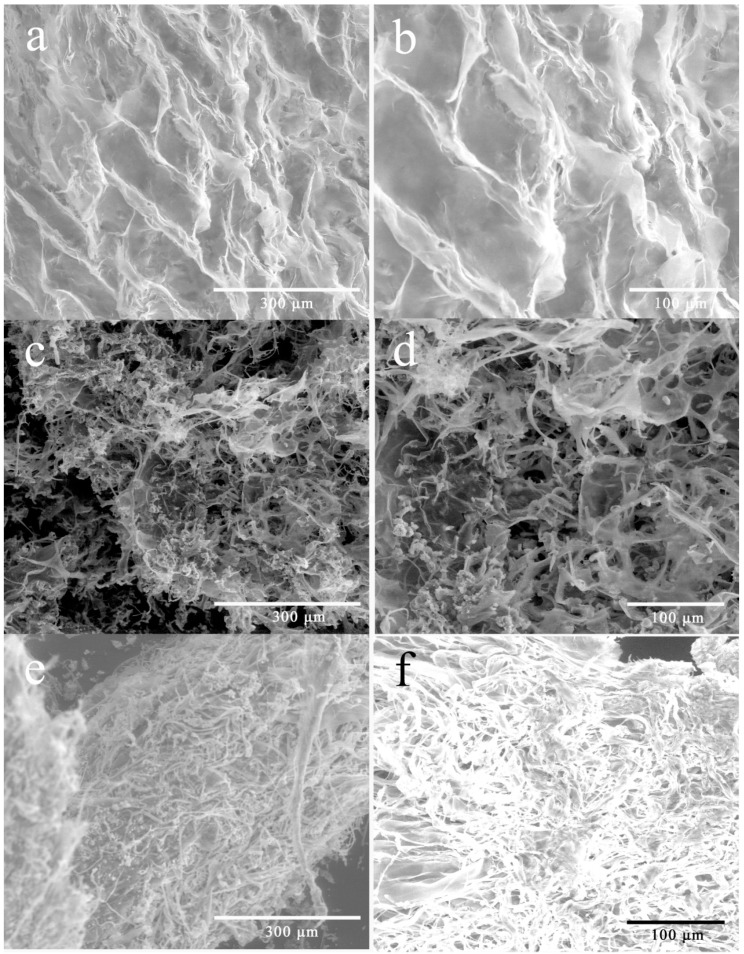
Environmental scanning electron microscopy images of WEP and its fractions. (**a**) WEP (×400); (**b**) WEP (×800); (**c**) WEP-0.3 (×400); (**d**) WEP-0.3 (×800); (**e**) WEP-0.4 (×400); (**f**) WEP-0.4 (×800).

The significant difference in solubility of WEP and its fractions may also relate to the morphology of pectin. As shown in Figure 4, WEP was relatively tidy with flake-like lamella (Figure 4a,b). However, the morphology of its fractions was significantly changed after fractionation. WEP-0.3 seemed to be sponge-like, where many pores appeared (Figure 4c,d). In the case of WEP-0.4, its morphology showed dense and compact rod-like shapes, and looked like noodles which were entangled and piled up with each other (Figure 4e,f). Polymer grain dissolution was progressed in three steps as stated by Parker et al. [27], including the entry of water through the grain surface to form a swollen gel layer, the release of polymer from the gel layer and the transfer of the released polymer chains into the bulk solution. With regard to WEP-0.4, grains were adhered to each other owing to its compact structure, which may obstruct the immersion of water and the disentanglement of the polymer chains from each other, considerably slowing the dissolution process despite mechanical stirring.

### 3.6. FT-IR Spectrum Characterization

**Figure 5:**
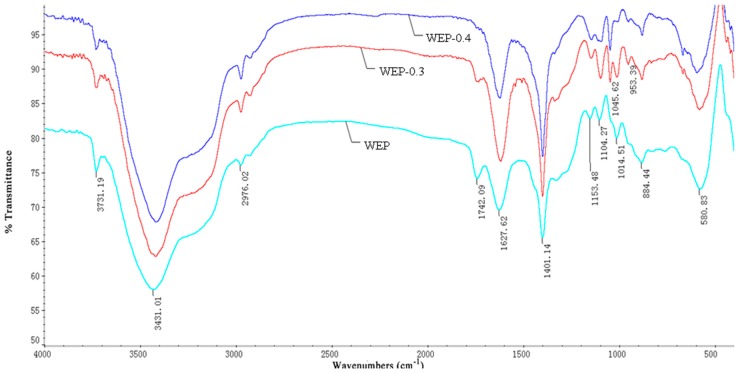
Fourier transform-infrared spectra of WEP and its fractions.

Properties of pectin such as solubility, crystalline structure and microscopic morphology may relate to the molecular structure. In order to further demonstrate the difference in solubility of WEP and its fractions, their FT-IR spectra were investigated. As Figure 5 displays, the FT-IR spectra of WEP and its fractions had no significant variations except for the bands at 1742 cm^−1^ and 1627 cm^−1^, which separately featured the presence of a methyl esterified carboxylic group and a free carboxylic group. The ratio of the peak area at 1740 cm^−1^ over the sum of the peak areas at 1740 and 1620 cm^−1^ is often used to calculate the DM [20]. In our study, the DMs of WEP, WEP-0.3 and WEP-0.4 were 27.58%, 10.46% and 6.67%, respectively, which was consistent with the results obtained from the titrimetric method, confirming that WEP-0.3 with a higher DM was eluted at a lower ionic strength (Figure 1). Generally, pectin with a higher DM should have a lower solubility due to the presence of higher levels of ester groups but a lower content of a free carboxyl groups in the pectin molecule [28]. Hence, WEP-0.4 ought to possess the highest solubility. Nevertheless, the observed phenomenon was the opposite, and the solubility of WEP-0.4 was the most inferior. A conjecture was that LMP with a lower DM was more susceptible to form gels by cross-linking with Ca^2+^, causing WEP-0.4 to have difficulty in dissolving. Therefore, the next step was to determine the mineral content of WEP and its fractions in order to validate the deduction.

### 3.7. Mineral Analysis

Table 2 displays the composition and contents of metals in WEP and its fractions. In our previous study, a considerably high content of Ca^2+^ was detected in water extracts from creeping fig seeds without precipitation and washing [13]. However, the content of Ca^2+^ in WEP was still high after successive precipitation and washing. The redundant Ca^2+^ was probably derived from the high Ca^2+^ in seeds, and tap water with a high Ca^2+^ content was used as dialysate during the initial stage. In comparison with significantly reduced level of magnesium, the levels of calcium were increased after dialysis. The content of Ca^2+^ in WEP-0.4 was significantly higher than that in WEP and WEP-0.3, indicating that WEP-0.4 had stronger binding abilities towards calcium. The low solubility of WEP-0.4 may be the consequence of microgel formation in the presence of a high content of Ca^2+^ and a lower DM. Hence, dialysate which contains multivalent metal ions such as Ca^2+^ should be avoided when LMP is dialyzed.

## 4. Conclusions

In the present work, two main fractions (WEP-0.3 and WEP-0.4) of crude water-extracted pectin (WEP) isolated from creeping fig seeds were obtained by fractionation using anion-exchange chromatography. The general physicochemical properties of WEP and its fractions were assayed and a significant difference in solubility of the two fractions was noticed. WEP-0.3 possessed a higher solubility than WEP, while WEP-0.4 became distinctly insoluble. An endeavor was made to explain the solubility difference of WEP-0.3 and WEP-0.4 from the point of crystalline structure, microscopic morphology and molecule structure. Nevertheless, the FT-IR spectra showed that WEP-0.4 had the lowest DM, which should lead to the highest solubility. The low solubility of WEP-0.4 was probably attributed to the considerably high calcium content and the lower DM of WEP-0.4 that was prone to forming microgels during dialysis. Therefore, a solution containing a metal divalent cationic was suggested to be forbidden for the dialysis of low methoxyl pectin.

## Figures and Tables

**Table 1:** Comparison of physicochemical properties of WEP and its fractions (WEP-0.3 and WEP-0.4).

Method	WEP	WEP-0.3	WEP-0.4
Color observation	Light yellow	Snow white	Snow white
Iodination reaction	(−) ^a^	(−)	(−)
Fehling’s test	(−)	(−)	(−)
FeCl_3_ reaction	(−)	(−)	(−)
Carbazole reaction	(+) ^b^	(+)	(+)
Peak at UV 280 nm	(+)	(−)	(−)
Coomassie brilliant blue reaction	(+)	(−)	(−)

^a^ Negative; ^b^ positive.

**Table 2:** Composition and amounts of metals in WEP and its fractions.

Samples	Metals(mg/g)				
	K	Na	Ca	Mg	Fe
Tap Water	2.59 ± 0.01 *	5.40 ± 0.02	4.68 ± 0.02	0.25 ± 0.00	0.01 ± 0.00
Deionzed Water	ND	ND	ND	ND	ND
WEP	0.23 ± 0.01 ^a^**	0.38 ± 0.01 ^a^	2.67 ± 0.01 ^a^	1.08 ± 0.00 ^a^	0.07 ± 0.01 ^a^
WEP-0.3	0.18 ± 0.02 ^a^	0.41 ± 0.00 ^a^	3.26 ± 0.03 ^b^	0.15 ± 0.01 ^b^	0.04 ± 0.01 ^a^
WEP-0.4	0.24 ± 0.01 ^a^	0.06 ± 0.01 ^b^	6.84 ± 0.03 ^c^	0.34 ± 0.01 ^c^	0.02 ± 0.02 ^a^

^a^ The data are the means of triplicate ± standard deviation. * The amount of metals in tap water is expressed as mg/L. ND represented no determination of metals. ** Different letters of the same line indicate that the means differ significantly (Tukey test, *p* < 0.05).

## References

[1] Taboada E., Fisher P., Jara R., Zúñiga E., Gidekel M., Cabrera J.C., Pereira E., Gutiérrez-Moraga A., Villalonga R., Cabrera G. (2010). Isolation and characterisation of pectic substances from murta (*Ugni molinae* Turcz) fruits. Food Chem..

[2] Grassino A.N., Barba F.J., Brnčić M., Lorenzo J.M., Lucini L., Brnčić S.R. (2018). Analytical tools used for the identification and quantification of pectin extracted from plant food matrices, wastes and by-products: A review. Food Chem..

[3] Ridley B.L., O’Neill M.A., Mohnen D. (2001). Pectins: Structure, biosynthesis, and oligogalacturonide-related signaling. Phytochemistry.

[4] Christiaens S., Uwibambe D., Uyttebroek M., Droogenbroeck B.V., Loey A.M.V., Hendrickx M.E. (2015). Pectin characterisation in vegetable waste streams: A starting point for waste valorisation in the food industry. LWT—Food Sci. Technol..

[5] Kumar A., Garg S., Mishra S.K., Champaign P. (2009). Immobilization of enzymes and biotechnological perspective. Biotechnology Applications.

[6] Marić M., Grassino A.N., Zhu Z., Brnčić M., Brnčić S.R. (2018). An overview of the traditional and innovative approaches for pectin extraction from plant food wastes and by-products: Ultrasound-, microwaves-, and enzyme-assisted extraction. Trends Food Sci. Technol..

[7] Shi X., Chang K., Schwarz J., Wiesenborn D., Shih M. (1996). Optimizing pectin extraction from sunflower heads by alkaline washing. Bioresour. Technol..

[8] Guo X., Han D., Xi H., Rao L., Liao X., Hu X., Wu J. (2012). Extraction of pectin from navel orange peel assisted by ultra-high pressure, microwave or traditional heating: A comparison. Carbohydr. Polym..

[9] El-Nawawi S., Heikal Y. (1995). Production of a low ester pectin by de-esterification of high ester citrus pectin. Carbohydr. Polym..

[10] Renard C.M., Thibault J.-F. (1996). Degradation of pectins in alkaline conditions: Kinetics of demethylation. Carbohydr. Res..

[11] Ralet M.-C., Dronnet V., Buchholt H.C., Thibault J.-F. (2001). Enzymatically and chemically de-esterified lime pectins: Characterisation, polyelectrolyte behaviour and calcium binding properties. Carbohydr. Res..

[12] Kim W., Smit C., Rao V. (1978). Demethylation of pectin using acid and ammonia. J. Food Sci..

[13] Liang R.-H., Chen J., Liu W., Liu C.-M., Yu W., Yuan M., Zhou X.-Q. (2012). Extraction, characterization and spontaneous gel-forming property of pectin from creeping fig (*Ficus pumila* Linn.) seeds. Carbohydr. Polym..

[14] Sun Y., Tang J., Gu X., Li D. (2005). Water-soluble polysaccharides from *Angelica sinensis* (Oliv.) Diels: Preparation, characterization and bioactivity. Int. J. Biol. Macromol..

[15] Dubois M., Gilles K.A., Hamilton J.K., Rebers P., Smith F. (1956). Colorimetric method for determination of sugars and related substances. Anal. Chem..

[16] Blumenkrantz N., Asboe-Hansen G. (1973). New method for quantitative determination of uronic acids. Anal. Biochem..

[17] Bradford M.M. (1976). A rapid and sensitive method for the quantitation of microgram quantities of protein utilizing the principle of protein-dye binding. Anal. Biochem..

[18] Monsoor M.A. (2005). Effect of drying methods on the functional properties of soy hull pectin. Carbohydr. Polym..

[19] Chen J., Liang R.-H., Liu W., Liu C.-M., Li T., Tu Z.-C., Wan J. (2012). Degradation of high-methoxyl pectin by dynamic high pressure microfluidization and its mechanism. Food Hydrocoll..

[20] Gnanasambandam R., Proctor A. (2000). Determination of pectin degree of esterification by diffuse reflectance Fourier transform infrared spectroscopy. Food Chem..

[21] Jie C., Sarma B., Evans J.M.B., Myerson A.S. (2011). Pharmaceutical Crystallization. Cryst. Growth Des..

[22] Whistler R.L. (1973). Solubility of Polysaccharides and Their Behavior in Solution.

[23] Agata Z., Cédric G., Alain B., Bruno P., Catherine G., Jean-Francois T., Marie-Christine R. (2007). Assessment of in vitro binding of isolated pectic domains to cellulose by adsorption isotherms, electron microscopy, and X-ray diffraction methods. Biomacromolecules.

[24] Sharma R., Kamboj S., Khurana R., Singh G., Rana V. (2015). Physicochemical and functional performance of pectin extracted by QbD approach from *Tamarindus indica* L. pulp. Carbohyd. Polym..

[25] Jiang Y., Du Y., Zhu X., Xiong H., Meng W.W., Hu J. (2012). Physicochemical and comparative properties of pectins extracted from *Akebia trifoliata* var. *australis* peel. Carbohyd. Polym..

[26] Kravtchenko T., Renoir J., Parker A., Brigand G. (1999). A novel method for determining the dissolution kinetics of hydrocolloid powders. Food Hydrocoll..

[27] Parker A., Vigouroux F., Reed W.F. (2000). Dissolution kinetics of polymer powders. AIChE J..

[28] Monsoor M., Proctor A. (2001). Preparation and functional properties of soy hull pectin. J. Am. Oil Chem. Soc..

